# Supplementary material for: Deletion of the von Hippel–Lindau gene causes sympathoadrenal cell death and impairs chemoreceptor-mediated adaptation to hypoxia
Source: EMBO Mol Med. 2014 Nov 10;6(12):1577–92. doi: 10.15252/emmm.201404153 (PMC4287976; doi:10.15252/emmm.201404153)
Supplement: Supplementary file 1 — Supplementary Information [file emmm0006-1577-sd1.pdf]

EMBO MOLECULAR MEDICINE

## SUPPLEMENTAL INFORMATION

# Deletion of the von Hippel-Lindau gene causes sympathoadrenal cell death and impairs chemoreceptor-mediated adaptation to hypoxia

**David Macías, M. Carmen Fernández-Agüera, Victoria Bonilla-Henao, and José López-Barneo**

## TABLE OF CONTENTS

**1. Extended Materials and Methods**

**2. Supplemental References**

**3. Supplemental Figures (S1-S6) and Legends**

**4. Supplemental Table.**

## 1. EXTENDED MATERIALS AND METHODS

### Mice Lines

Catecholaminergic-specific *Vhl* ablation or *Hif-1*$\alpha$ and/or *Hif-2*$\alpha$ over expression was achieved by mating mice with these alleles with a Th-IRES-Cre transgenic mouse line to generate the experimental TH-VHL$^{KO}$, TH-HIF1$^{dPA}$, TH-HIF2$^{dPA}$ and TH-HIF1$^{dPA}$; TH-HIF2$^{dPA}$ mouse lines, respectively. Age-matched littermates with same *Vhl*$^{flox/flox}$, *Vhl*$^{flox/-}$, *HIF1AdPA*$^{flox/+}$ and *HIF2AdPA*$^{flox/+}$ genotypes but lacking Cre recombinase were used as controls. Since no differences between *Vhl*$^{flox/flox}$ and *Vhl*$^{flox/-}$ genotypes were found for the phenotypes tested, both genotypes were considered as a single control group (VHL$^{WT}$). TH-VHL$^{KO}$ mice were subsequently mated with *Phd3* null animals to generate the control VHL$^{WT}$;PHD3$^{KO}$ (lacking Cre recombinase) and experimental TH-VHL$^{KO}$;PHD3$^{KO}$ mouse lines. For catecholaminergic-specific *Vhl* deletion in adult mice, *Vhl*$^{flox/-}$ animals were bred with Th-IRES-Cre$^{ER}$ transgenic mouse line to obtain the experimental TH-CRE$^{ER}$-VHL$^{KO}$ mice. VHL$^{WT}$ and TH-CRE$^{ER}$-VHL$^{KO}$ two-month-old littermates were fed with a tamoxifen (TMX) diet (TAM400/CreER, 400 mg tamoxifen citrate per kg diet, Harlan

laboratories) for a month and then fed back with normal diet (Teklad global diet, Harlan laboratories). To verify the $Cre^{ER}$-mediated conditional excision efficiency in the carotid body (CB) and adrenal medulla (AM) after TMX treatment, we mated Th-IRES-$Cre^{ER}$ animals with R26R-STOP-LacZ reporter mouse strain (Soriano, 1999). The genetic background for all the mouse strains used in this study was C57BL/6. Unless indicated in the figure legends, adult 2 to 3 month-old-animals from both genders were used for the experiments.

**Immunohistochemistry**

TH and GFAP were detected with the rabbit anti-mouse TH (1:5000, Novus, NB300-109) and rabbit anti-mouse GFAP (1:500, Dako, 70334) polyclonal antibodies, respectively. The neuronal marker HuC/D was detected with mouse anti-human HuC/D (1:100, Molecular Probes, A-21271) after antigen retrieval in 10 mM sodium citrate buffer, pH= 6. BrdU administration and staining was performed as previously reported (Pardal *et al*., 2007) using a rat anti-BrdU (1:100, Accurate Chemicals). For 3,3- diaminobenzidine-based detection, an $Envision^{+}$ kit (Dako) was used according to the manufacturer's recommended protocol. For fluorescence detection, Alexa-Fluor 488- and 568-conjugated anti-rabbit IgG (1:500, Molecular Probes), Alexa-Fluor 568-conjugated anti-mouse IgG (1:500, Molecular Probes) and fluorescein isothiocyanate (FITC)-conjugated anti-rat IgG (1:200, Jackson Immunoresearch) secondary antibodies were used. X-gal staining of 50 μm thick CB and AM slices was carried out as described previously (Villadiego *et al.,* 2005).

**Electron Microscopy**

$VHL^{WT}$ and TH-$VHL^{KO}$ mice were anesthetized and intracardially perfused with a PBS-based fixative solution containing 4% paraformaldehyde plus 2.5% glutaraldehyde (Electron Microscopy Sciences). Carotid bifurcations were extracted and maintained for 2 hours in the same fixative. Next, carotid bifurcations were embedded in gelatin and 100 μm thick slices were obtained with a vibratome (Leica VT 1000S). Those sections with CB were embedded for transmission electron microscopy. Briefly, sections were postfixed with 1% osmium tetroxide for 1 hour (4ºC), rinsed with cacodilate buffer, stained with 2% uranyl acetate, dehydrated in acetone series, embedded in Spurr resin (Tedpella) and polymerized in an oven at 70ºC for 7 hours. Semithin sections (200nm thickness) were obtained with a glass

knife and stained with 1% toluidine blue for glomeruli localization and reorientation using a conventional optic microscope. Once a suitable block face of the selected area was trimmed, several ultrathin sections (70nm) were obtained using an ultramicrotome (Leica UC7) equipped with diamond knife (Diatome) and collected on 200 mesh cupper grids.

**Generation and Differentiation of Carotid Body Neurospheres**

Carotid bodies (CBs) were dissected and enzymatically dispersed in 3 ml of phosphate buffered saline (PBS) containing 0.8 mg trypsin (Sigma), 1.8 mg collagenase II (Sigma), 3.75U elastase (Calbiochem) and 0.05 mM $CaCl_2$ in a shaker (550 rpm) at 37ºC for 15 minutes. Subsequently, the tissue was mechanically dissociated by pipetting, and incubated for 5 minutes at 37ºC without shaking. The resulting CB cells suspension was cultured with neural crest (NC) medium as previously published in our laboratory (Pardal *et al.*, 2007) with the 15% chick embryo extract replaced with 20% bovine foetal serum (FBS). All neurosphere cultures were maintained at 3% $O_2$, 5% $CO_2$ and 37ºC for 8 days. Neurosphere culture medium was supplemented with 0.5 mM DMOG from day 5 where indicated. Once formed, the number and diameter of neurospheres were measured using ImageJ software on bright field photographs (Olympus IX71). For immunocytochemical identification of cells within the neurospheres, these were either collected, fixed with formalin solution (Sigma) for 30 minutes, cryopreserved (30% sucrose) and embedded for cryosectioning (10 $\mu$m thick), or replated onto fibronectin-treated coverslips and cultured for 3 or 10 days with DMEM/F12 (Gibco) medium supplemented with 10% FBS, 1% penicillin/streptomycin, 1% L-Glutamine, 1% N2 (Gibco), 1% B27 (Gibco), 0.7 U/mL insulin, 0.15 mg/mL suramin (Sigma) and 1 $\mu$M cAMP to improve differentiation. Subsequently, both neurosphere sections and flat neurosphere colonies were stained following standard procedures. The primary antibodies used were: rabbit anti-mouse TH (1:5000, Novus, NB300-109), mouse anti-mouse/rat Nestin (1:500, Millipore, MAB353) and mouse anti-mouse Sma-$\alpha$ (1:300, Sigma, A2547), followed by the same secondary antibodies described above.

**PCR Analyses**

To evaluate the knockdown efficacy of each *Phd3, Hif-1*$\alpha$ *or Hif-2*$\alpha$ shRNA LVs,

mouse embryonic fibroblast cells (NIH3T3) were transduced at a multiplicity of infection (MOI) of 5 and analyzed by qRT-PCR. Total mRNA was isolated with TRIzol (Invitrogen) following the manufacturer's instructions. Reverse transcription of mRNA was performed with a Superscript II reverse transcription kit (Invitrogen), and *Phd3* cDNA was amplified using the primers:

mPHD3-F 5´-CAGACCGCAGGAATCCACAT-3´;

mPHD3-R 5´-CATCGAAGTACCAGACAGTCATAGC-3´;

mHIF-1$\alpha$-F 5'-GAAACGACCACTGCTAAGGCA-3';

mHIF-1$\alpha$-R 5'- GGCAGACAGCTTAAGGCTCCT-3';

mHIF-2$\alpha$-F 5'- CAACCTGCAGCCTCAGTGTATC-3';

mHIF2 $\alpha$-R 5'- CACCACGTCGTTCTTCTCGAT-3'

and SYBR Green Universal Master Mix (Applied Biosystems) with a 7500 Fast Real-Time PCR System (Applied Biosystems). The *Arbp* housekeeping gene was used for normalization. For the testing of floxed allele excision in brain catecholaminergic tissues, genomic DNA from the ventral mesencephalon was isolated with DNeasy blood and tissue kit (Qiagen) following the manufacture's protocol. DNA extracted from cells in the tail was used as control. Conditional (*2-lox*) and excised (*1-lox*) alleles were amplified by standard PCR with specific primers as previously described (Haase *et al*., 2001).

## 2. SUPPLEMENTAL REFERENCES


Haase, V.H., Glickman, J.N., Socolovsky, M., and Jaenisch, R. (2001). Vascular tumors in livers with targeted inactivation of the von Hippel-Lindau tumor suppressor. Proc. Natl. Acad. Sci. U. S. A. *98*, 1583–1588.

Pardal, R., Ortega-Sáenz, P., Durán, R., and López-Barneo, J. (2007). Glia-like stem cells sustain physiologic neurogenesis in the adult mammalian carotid body. Cell *131*, 364–377.

Soriano, P. (1999). Generalized lacZ expression with the ROSA26 Cre reporter strain. Nat. Genet. *21*, 70–71.

Villadiego, J., Méndez-Ferrer, S., Valdés-Sánchez, T., Silos-Santiago, I., Fariñas, I., López-Barneo, J., and Toledo-Aral, J.J. (2005). Selective glial cell line-derived neurotrophic factor production in adult dopaminergic carotid body

cells in situ and after intrastriatal transplantation. J. Neurosci. *25*, 4091–4098.

## 3. SUPPLEMENTAL FIGURES

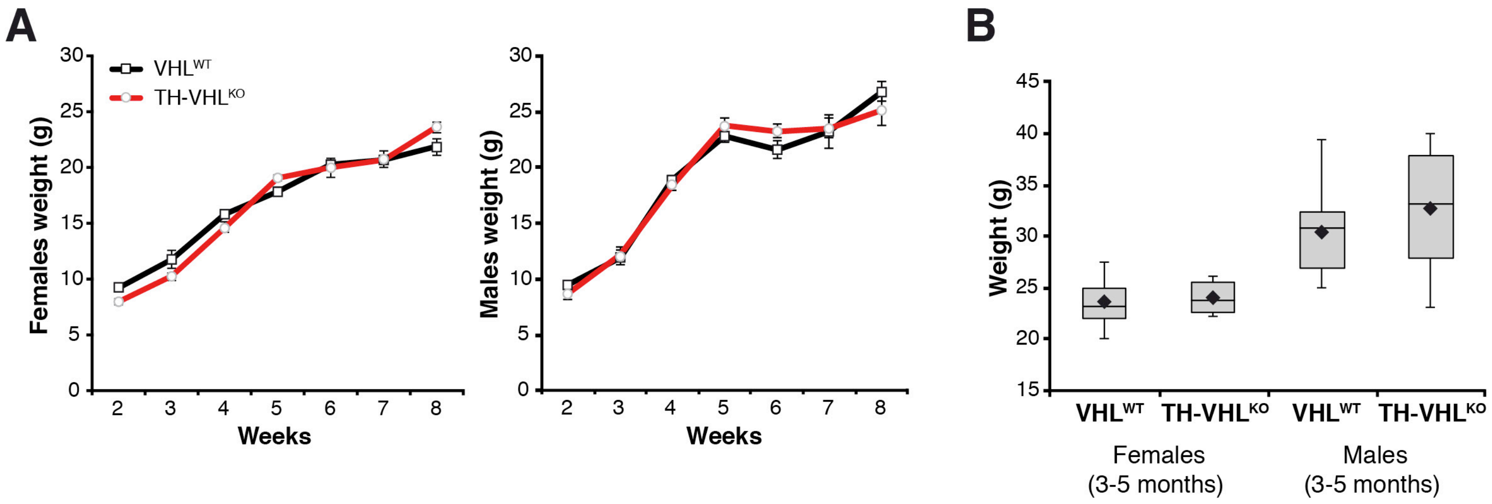


**Figure S1.**

**Body weight of VHL$^{WT}$ and TH-VHL$^{KO}$ mice.** (**A**) Time course of gain of weight in VHL$^{WT}$ and TH-VHL$^{KO}$ mice during the first 8 postnatal weeks (n = 5 per genotype and sex). (**B**) Box diagrams illustrating the weight distribution of adult (aged 3 to 5 months) VHL$^{WT}$ and TH-VHL$^{KO}$ mice (n = 13 per genotype and sex).

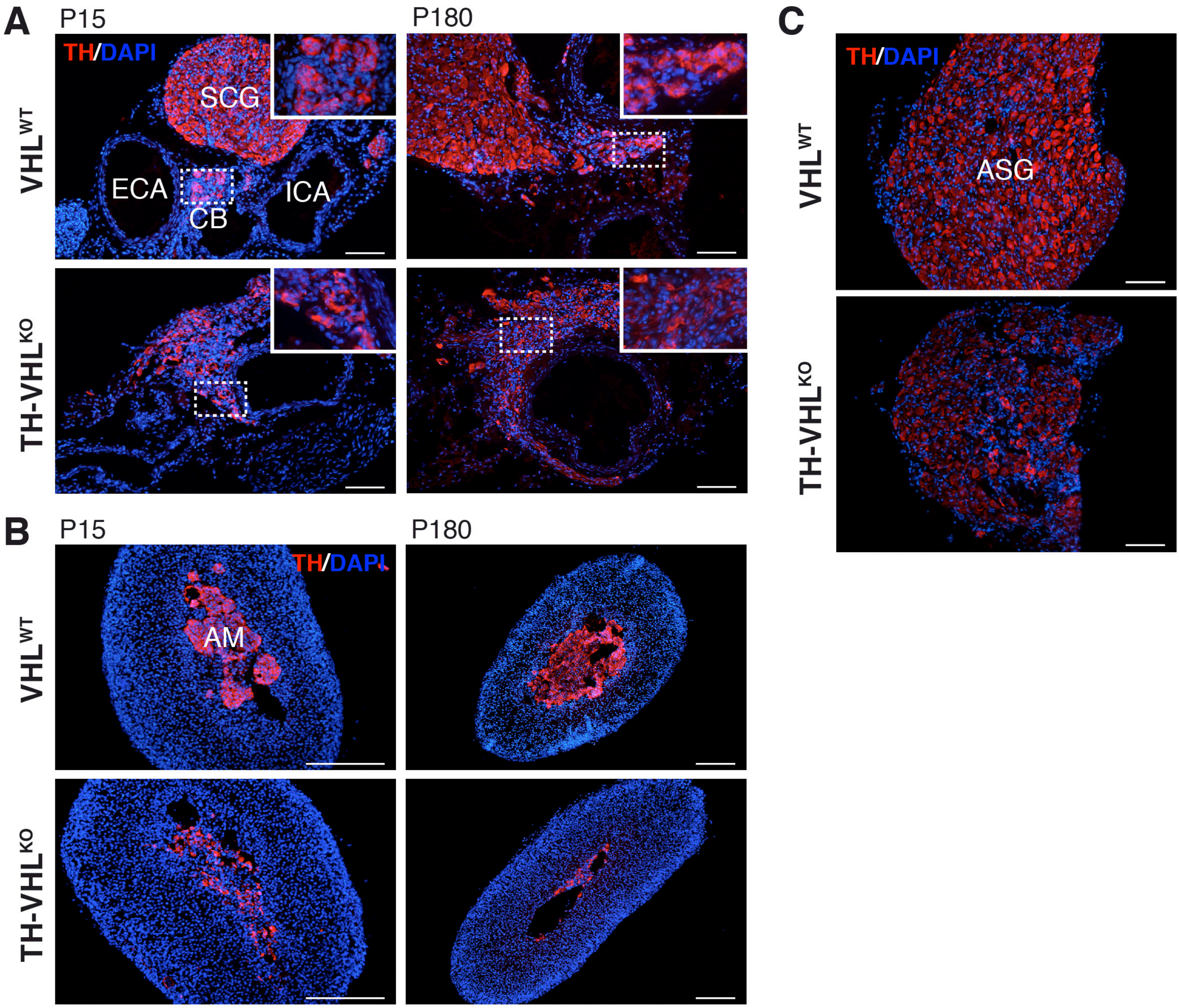


**Figure S2**. Related to Figure 1.

**Atrophy of sympathoadrenal organs in TH-VHL$^{KO}$ mice.** (**A** and **B**) Histological alterations found in the carotid bifurcation (**A**) and adrenal gland (**B**) of TH-VHL$^{KO}$ mice compared with VHL$^{WT}$ at the indicated postnatal age (in days). The areas inside the rectangles in **A** are shown in the insets at higher magnification. ECA, external carotid artery; ICA, internal carotid artery; CB, carotid body; SCG, superior cervical ganglion; AM, adrenal medulla. Scale bars: (**A**) 100 μm; (**B**) 200 μm. (**C**) TH immunodetection illustrating cell loss in the abdominal sympathetic ganglia of TH-VHL$^{KO}$ compared with VHL$^{WT}$ mice (8-12 weeks of age). ASG, Abdominal sympathetic ganglia. Scale bars: 100 μm.

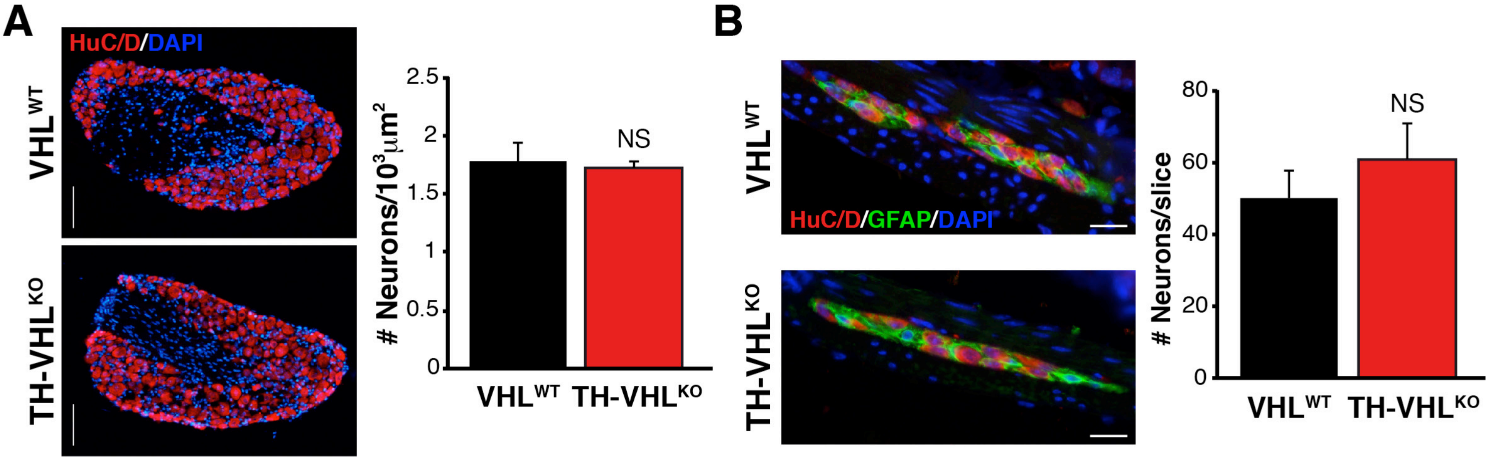


**Figure S3.** Related to Figure 1.

**Peripheral non-catecholaminergic neurons unaffected in TH-VHL$^{KO}$ mice.** (**A** and **B**) Immunostaining of neurons in the dorsal root ganglia (**A**) and myenteric plexus (**B**) with the neuronal marker HuC/D+ illustrating that these organs are unaltered in TH-VHL$^{KO}$ mice compared with controls. Scale bars: (**A**) 100 μm; (**B**) 20 μm. Cell counting was performed on 6 animals (8-12 weeks old) per genotype. NS, non-significant (unpaired two-tailed *t* test).

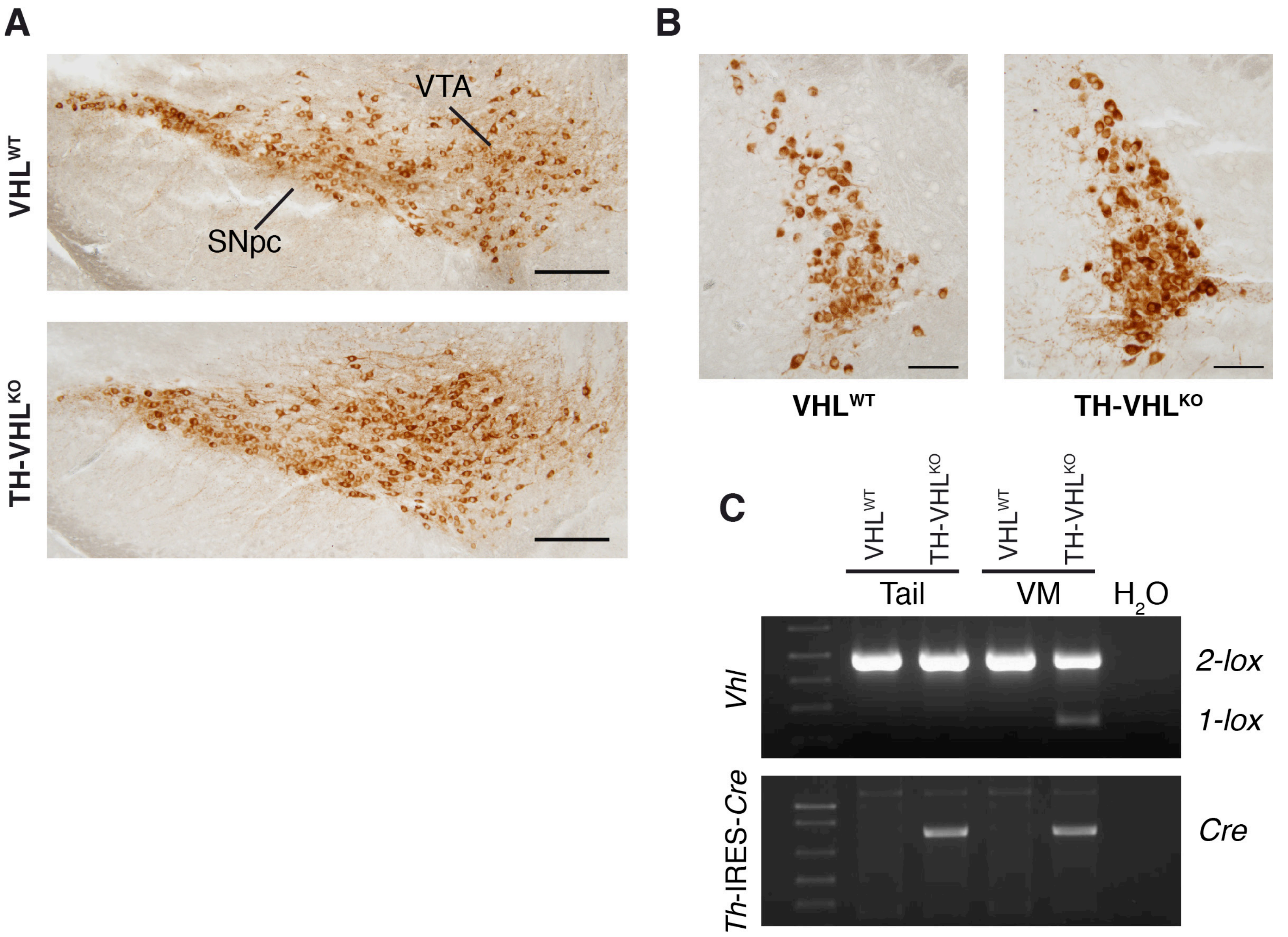


**Figure S4.** Related to Figure 1.

**Central catecholaminergic neurons unaffected in juvenile TH-VHL$^{KO}$ mice.** (**A** and **B**) TH immunostaining of mesencephalic dopaminergic neurons (**A**) and noradrenergic neurons in the locus coeruleus (**B**) of VHL$^{WT}$ and TH-VHL$^{KO}$ mice (10 weeks of age). Scale bars: 200 μm. (**E**) PCR analysis of genomic DNA isolated from tail and ventral mesencephalon (VM) with specific primers amplifying the floxed (*2-lox*) and excised (*1-lox*) *Vhl* allele. Recombination of the floxed *Vhl* allele was only detected in the VM of TH-VHL$^{KO}$ (Th-IRES-Cre-positive) mice, but not in the tail.

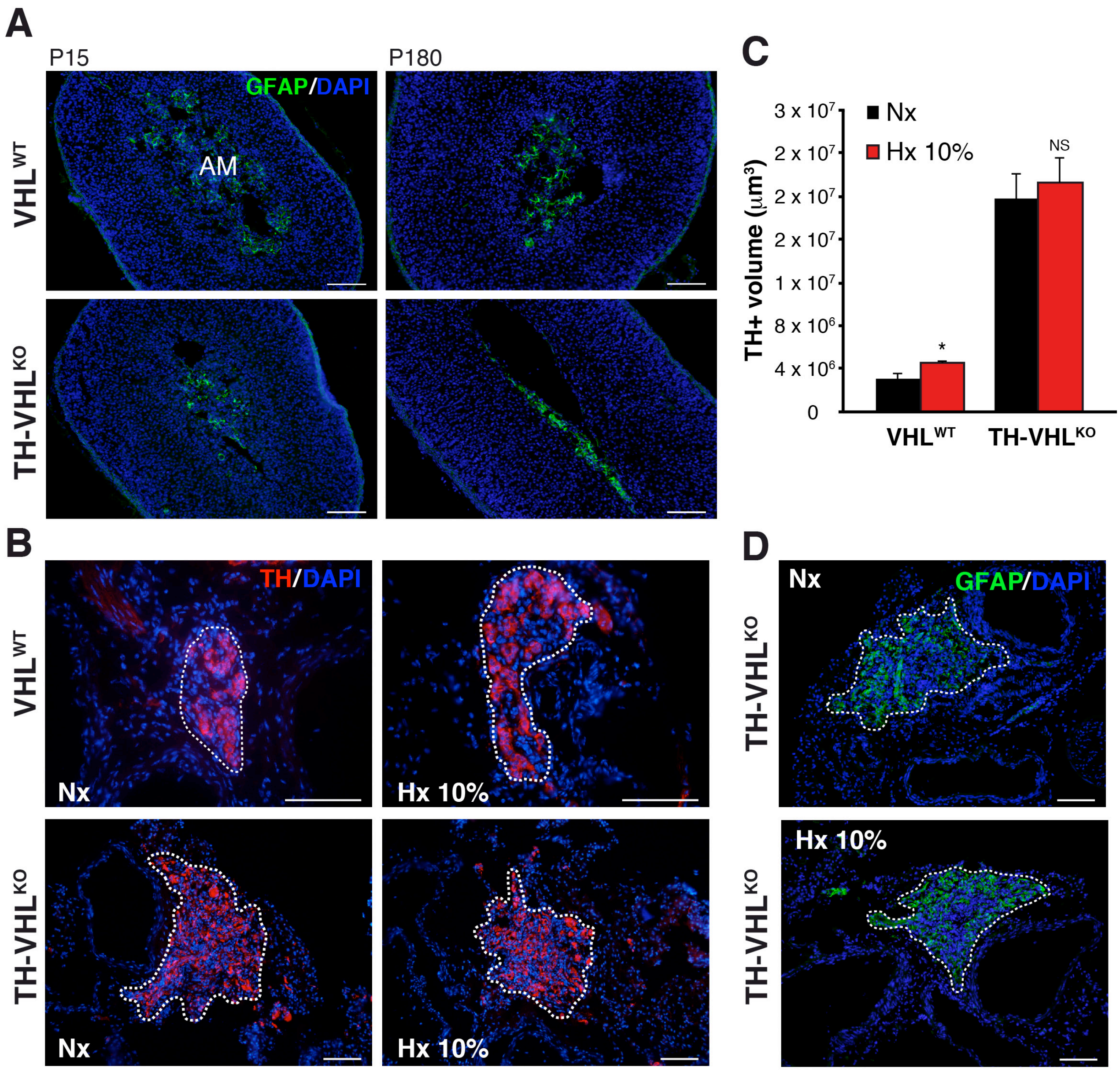


**Figure S5.** Related to Fig. 3

**Immunohistochemical analysis of GFAP+ cells in adrenal medulla in VHL$^{WT}$ and TH-VHL$^{KO}$ mice and carotid body growth in chronic hypoxia.** (**A**) GFAP+ cells in the adrenal medulla. AM, adrenal medulla. Scale bars: 100 μm. (**B**) Representative immunofluorescence photographs showing the appearance of the TH+ region (circumscribed by the dotted white lines) in the adult CB (VHL$^{WT}$ mice) and CB-SCG area (TH-VHL$^{KO}$) in response to chronic hypoxia (7 days at 10% $O_2$). Scale bars: 100 μm. (**C**) Quantification of the TH+ volume in VHL$^{WT}$ and TH-VHL$^{KO}$ animals (8 weeks of age) exposed to chronic hypoxia (10% $O_2$) for 7 days (n = 3 per genotype and condition). *P = 0.047 (unpaired two-tailed *t* test). NS (non-significant). Nx, normoxia; Hx, hypoxia. (**D**). GFAP+ area in TH-VHL$^{KO}$ mice exposed to normoxia and hypoxia.

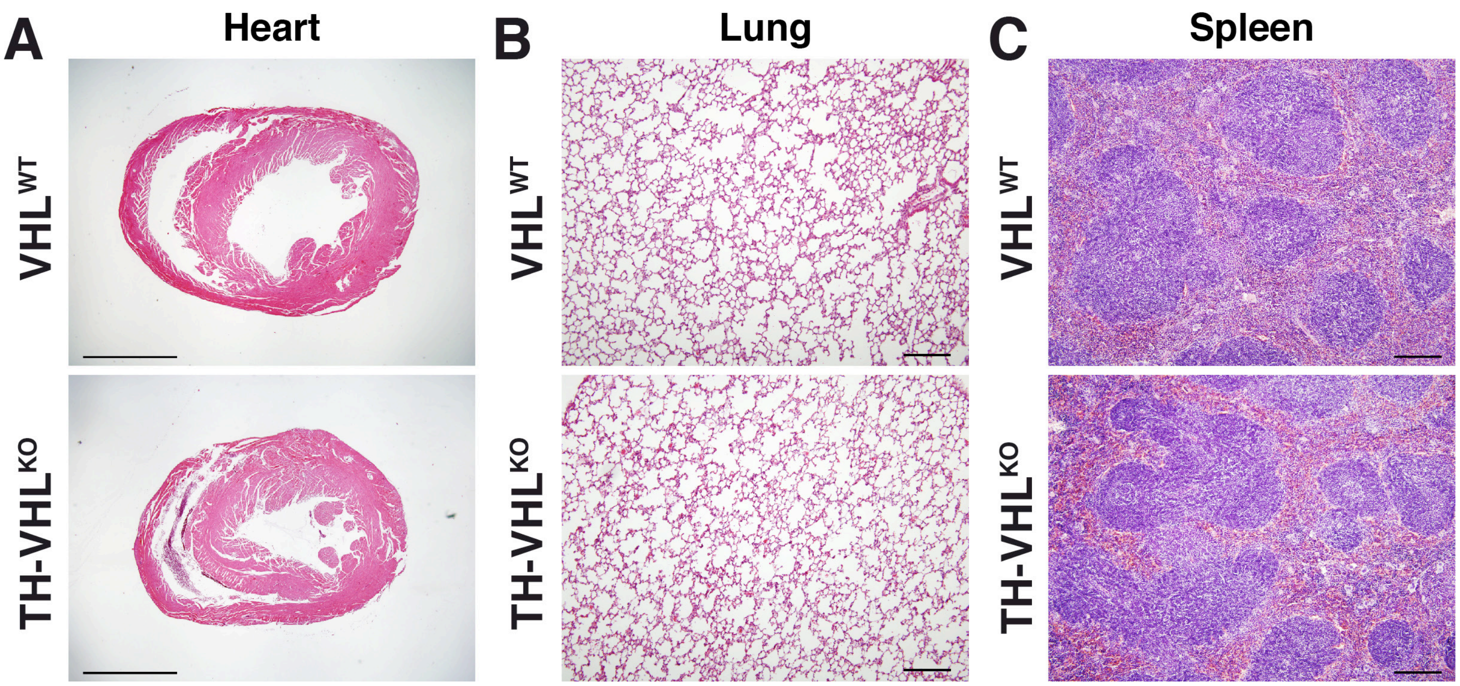


**Figure S6.** Related to Fig. 9.

**Histopathology of TH-VHL$^{KO}$ mice maintained in normoxia**. (**A**-**C**) Hematoxylin and eosin staining of the heart (**A**), lung (**B**) and spleen (**C**) of VHL$^{WT}$ and TH-VHL$^{KO}$ mice maintained in a normoxic atmosphere (n = 6 per genotype). Scale bars: (**A**) 2 mm; (**B** and **C**) 200 μm. All analyses were performed with 8-12 weeks old mice.

## 4. SUPPLEMENTAL TABLE

Table S1. Respiratory parameters.

| Parameter | VHL$^{WT}$ | TH-VHL$^{KO}$ | VHL$^{WT}$;PHD3$^{KO}$ | TH-VHL$^{KO}$;PHD3$^{KO}$ |
|---|---|---|---|---|
| | Nx / Hx | Nx / Hx | Nx / Hx | Nx / Hx |
| Ti (msec) | 140.4 ± 3.1 / 101.7 ± 4.4[a] | 143.8 ± 7.4 / 142.2 ± 1.2[NS] | 137.1 ± 15.1 / 104.3 ± 5.4[NS] | 152.8 ± 3.8 / 163.5 ± 10.3[NS] |
| Te (msec) | 308.8 ± 22.3 / 161.5 ± 6.2[b] | 346.3 ± 25.5 / 267.5 ± 23.5[NS] | 343.9 ± 9.6 / 180.5 ± 10.3[e] | 489.9 ± 59.1 / 495.1 ± 76.1[NS] |
| PIF (ml/s) | 2.28 ± 0.23 / 3.63 ± 0.57[NS] | 2.83 ± 0.30 / 2.39 ± 0.29[NS] | 2.85 ± 0.16 / 4.03 ± 0.26[f] | 2.43 ± 0.13 / 2.77 ± 0.26[NS] |
| PEF (ml/s) | 1.97 ± 0.12 / 2.84 ± 0.36[NS] | 2.18 ± 0.08 / 2.46 ± 0.17[NS] | 1.83 ± 0.10 / 2.77 ± 0.17[g] | 1.83 ± 0.17 / 2.91 ± 0.43[NS] |
| TV (ml) | 0.19 ± 0.01 / 0.22 ± 0.02[NS] | 0.24 ± 0.01 / 0.22 ± 0.02[NS] | 0.22 ± 0.01 / 0.24 ± 0.01[NS] | 0.21 ± 0.01 / 0.26 ± 0.03[NS] |
| RT (msec) | 195.9 ± 20.9 / 107.22 ± 4.5[c] | 243.8 ± 20.3 / 183.9 ± 17.0[NS] | 253.8 ± 9.6 / 126.4 ± 6.3[h] | 392.1 ± 63.2 / 242.0 ± 28.4[NS] |
| MV (ml) | 28.63 ± 3.48 / 54.13 ± 7.78[d] | 32.44 ± 2.73 / 34.89 ± 3.76[NS] | 30.52 ± 1.21 / 54.57 ± 5.12[i] | 24.54 ± 1.28 / 30.19 ± 4.51[NS] |

All values are means ± SEM. Ti, inspiratory time. Te, expiratory time. PIF, peak inspiratory flow. PEF, peak expiratory flow. TV, tidal volume, RT, relaxation time. MV, minute volume. Number of animals per genotype n = 5. a (P = 0.0003); b (P = 0.0007); c (P = 0.006); d (P = 0.02); e (P = 0.00002); f (P = 0.008); g (P = 0.03); h (P = 0.00003); i (P = 0.003). NS, non-significant. All comparisons were assessed by unpaired two-tailed *t* test. Nx, normoxia. Hx, hypoxia.
